# Whole‐Exome Sequencing in Undiagnosed Muscular Dystrophies: A High Diagnostic Yield and Novel Insights From Iranian Families

**DOI:** 10.1155/humu/6592066

**Published:** 2026-08-03

**Authors:** Nasibeh Soltani, Zahra Shahbazi, Mohammad Sadegh Fallah, Morteza Karimipoor, Hamideh Bagherian, Samira Dabbagh Bagheri, Tina Shirzadeh, Fatemeh Zafarghandi Motlagh, Gelareh Rabie Salehi, Shahrzad Younesikhah, Sadaf Asnavandi, Razie Zeinali, Ziba Majidi, Sirous Zeinali

**Affiliations:** ^1^ Molecular Medicine Department, Biotechnology Research Center, Pasteur Institute of Iran, Tehran, Iran, pasteur.ac.ir; ^2^ Department of Medical Laboratory Science, School of Allied Medical Sciences, Tehran University of Medical Sciences, Tehran, Iran, tums.ac.ir; ^3^ Basic and Molecular Epidemiology of Gastrointestinal Disorders Research Center, Research Institute for Gastroenterology and Liver Diseases, Shahid Beheshti University of Medical Sciences, Tehran, Iran, sbmu.ac.ir; ^4^ Kawsar Human Genetics Research Center, Tehran, Iran

**Keywords:** autosomal recessive inheritance, consanguinity, genetic diagnosis, muscular dystrophies, novel variants, whole-exome sequencing (WES)

## Abstract

**Background:**

Muscular dystrophies (MDs) are a genetically heterogeneous group of disorders, posing significant diagnostic challenges, especially in populations with high consanguinity. Despite advances in genetic testing, a substantial proportion of patients remain undiagnosed. Whole‐exome sequencing (WES) has emerged as a powerful tool for identifying causal variants in such unresolved cases. To identify the genetic basis of nondystrophinopathic MDs in Iranian families with inconclusive prior genetic testing and to evaluate the diagnostic yield and mutational spectrum in this population.

**Methods:**

We performed WES on one affected individual from each of 10 unrelated Iranian families with clinically diagnosed MD. Candidate variants were prioritized based on in silico prediction tools (SIFT, PolyPhen‐2, CADD, SpliceAI), population frequency databases (gnomAD, 1000 Genomes, EVS), and ACMG/AMP guidelines. Findings were validated by Sanger sequencing, MLPA, and STR haplotype analysis. Segregation analysis was performed in available family members.

**Results:**

WES led to a diagnostic yield of 69.2% (9/13 variants in 10 families) after segregation analysis and ACMG‐based reclassification. We identified 13 candidate variants in 10 known MD‐associated genes, including *DYSF*, *SGCA*, *TK2*, *MAP3K20*, *LMNA*, *COL6A1*, *COL6A2*, *ITGA7*, *MICU1*, and *SGCB*. Among these, seven variants (54%) were novel. The majority of cases (84.6%) followed an autosomal recessive pattern, consistent with high parental consanguinity (70%). Notably, a de novo splice‐site variant in COL6A2 (c.1053+1G>T) was identified in a sporadic case, confirming an autosomal dominant inheritance. Challenges in variant interpretation were observed in families with variants in tightly linked genes (*COL6A1* and *COL6A2*), highlighting the role of linkage disequilibrium in founder populations.

**Conclusion:**

WES is a highly effective diagnostic strategy for genetically heterogeneous MDs, particularly in consanguineous populations. Our study expands the mutational spectrum of MDs in Iran and provides critical data for genetic counseling, prenatal diagnosis, and future therapeutic development. The high rate of novel variants underscores the importance of population‐specific genomic studies.

## 1. Introduction

Muscular dystrophies (MDs) are a heterogeneous group of genetically inherited degenerative disorders characterized by progressive muscle weakness and atrophy. The clinical spectrum ranges from partial impairment of mobility to severe disability, significantly affecting quality of life and life expectancy [1]. A dystrophic pattern on muscle biopsy—marked by muscle fiber degeneration, necrosis, and fibrosis—is a hallmark feature observed across various genetically defined conditions associated with progressive muscle degeneration [2, 3]. Despite their diverse clinical and molecular underpinnings, all forms of MD share common myopathic morphological features [4].

Elevated serum levels of creatine kinase (CK) or creatine phosphokinase (CPK), an intracellular enzyme highly expressed in skeletal muscle, often precede the onset of clinical symptoms and serve as a key biomarker for muscle damage [5, 6]. Furthermore, significant phenotypic variability—including differences in age of onset, disease severity, progression, and prognosis—is commonly observed even among individuals with the same genetic mutation, reflecting pronounced genotype–phenotype heterogeneity [7, 8]. Given the overlapping clinical presentations and variable expressivity across MD subtypes, establishing a precise diagnosis remains a major challenge. This underscores the necessity for reliable diagnostic strategies that enable effective genetic counseling, prenatal diagnosis, and, where possible, early therapeutic intervention [9, 10].

Conventional molecular diagnostic approaches for genetically heterogeneous disorders like MDs are often time‐consuming, costly, and inefficient—particularly when multiple genes can cause similar phenotypes. Despite advances in clinical diagnostics, the genetic basis of many MD cases remains unidentified. Whole‐exome sequencing (WES) has emerged as a powerful, high‐throughput tool capable of rapidly uncovering the molecular basis of monogenic diseases by targeting the protein‐coding regions of the genome, which harbor approximately 85% of known disease‐causing variants. In many cases, WES is the only method capable of identifying the causative gene or mutation, especially in patients with atypical or undiagnosed presentations [11, 12].

In Iran, several studies have been conducted to characterize the genetic landscape of MDs. These investigations have primarily identified large deletions and point mutations, often using Sanger sequencing [13, 14]. For instance, Haghshenas et al. [15] evaluated point mutations in the dystrophin gene among Iranian patients with Duchenne and Becker MD. In 2020, Zamani et al. [16] published the first comprehensive cohort study of Duchenne MD in the Iranian population, defining the mutation spectrum in 314 patients and reporting two novel nonsense mutations.

Over the past decade, our research team has conducted systematic studies on MDs in Iran, leading to the identification of novel mutations and disease patterns [17–20]. In 2020, a collaborative cohort study genetically characterized 112 limb‐girdle muscular dystrophy (LGMD) patients and investigated potential founder effects [14].

Despite these advances, a significant proportion of Iranian patients with nondystrophinopathic MDs remain undiagnosed at the molecular level. The present study is aimed at bridging this gap by applying WES in a cohort of 10 Iranian families with unresolved diagnoses.

## 2. Methods

### 2.1. Patient and Family Recruitment

The study included 10 unrelated families with a total of 27 affected individuals (16 females, 59.3%; 11 males, 40.7%) exhibiting heterogeneous clinical manifestations. Families were referred to Dr. Zeinali′s Medical Genetics Laboratory at the Kawsar Human Genetics Research Center, Tehran, Iran (https://www.ncbi.nlm.nih.gov/gtr/labs/506443/), the Pasteur Institute of Iran, and Mofid Children′s Hospital for clinical evaluation and genetic testing. Experienced neurologists and genetic counselors supervised data collection, including demographic and clinical features, parental consanguinity, family history, histological findings, electromyography (EMG) results, serum CK levels, mode of inheritance, signs and symptoms, and disease onset and progression. Data were recorded using standardized questionnaires. Pedigrees were drawn using Progeny 9 software (https://progenygenetics.com/).

Inclusion criteria were presence of at least two affected individuals per family (except for one sporadic case, explained in Section 3), preferably an affected female, and elevated serum CK levels. Exclusion criteria included a confirmed diagnosis of dystrophinopathy.

Peripheral blood samples were collected from patients and family members. Written informed consent was obtained from all parents or legal guardians. The study protocol was approved by the Ethics Committee of the Pasteur Institute of Iran.

In the diagnostic process, biochemical parameters (lactate dehydrogenase [LDH], CK, alanine aminotransferase [ALT], aspartate aminotransferase [AST], and aldolase), echocardiography, and MRI were used when available and clinically indicated. The possibility of *SMN1* gene deletions or mutations (causing spinal muscular atrophy with DMD‐like symptoms) was ruled out using Sanger sequencing or multiple ligation probe amplification (MLPA).

### 2.2. Pre‐WES Diagnostic Workflow and First‐Tier Testing

Prior to WES, all patients underwent a stepwise diagnostic evaluation. Dystrophinopathies (Duchenne/Becker MD) were first excluded using MLPA and/or Sanger sequencing of the DMD gene. Subsequently, targeted molecular testing for LGMD was performed, which included (i) linkage analysis using polymorphic STR markers in consanguineous families to identify candidate homozygous regions; (ii) Sanger sequencing of common LGMD‐associated genes (e.g., *CAPN3*, *DYSF*, *SGCA*, *SGCB*, *SGCG*, and *SGCD*) within linked regions or based on clinical phenotype; and (iii) MLPA for detection of large deletions/duplications in sarcoglycan genes when clinically indicated. WES was reserved for cases in which these targeted approaches failed to identify a causative variant or yielded inconclusive results. For newly referred families, first‐tier clinical and laboratory assessments (serum CK/CPK, EMG/NCV, and muscle biopsy with immunohistochemistry when available) were used to characterize the myopathic phenotype and guide pre‐WES gene prioritization.

### 2.3. Exome Sequencing, Data Analysis, and Interpretation

Genomic DNA was extracted from peripheral blood leukocytes using the proteinase K method [21]. WES was performed on the Illumina HiSeq platform. Data analysis followed a previously published pipeline [22]. Candidate variants were prioritized based on in silico prediction tools:

• Sorting Intolerant From Tolerant (SIFT) (https://sift.bii.a-star.edu.sg/)
• Polymorphism Phenotyping v2 (PolyPhen‐2) (http://genetics.bwh.harvard.edu/pph2/)
• MutationTaster (https://www.mutationtaster.org/)
• Functional Analysis through Hidden Markov Models v2.3 (fathmm) (http://fathmm.biocompute.org.uk/)
• Protein Variation Effect Analyzer (PROVEAN) (https://www.jcvi.org/)
• Combined Annotation Dependent Depletion (CADD) (https://cadd.gs.washington.edu/)
• Human Splicing Finder (http://umd.be/Redirect.html)
• Varsome (https://varsome.com/), a platform integrating multiple prediction algorithms.

Synonymous variants and those with a minor allele frequency (MAF) > 1% in the National Heart, Lung, and Blood Institute (NHLBI) Exome Variant Server (http://evs.gs.washington.edu/EVS/) and 1000 Genomes Project (https://www.internationalgenome.org/) were excluded. Variants were classified according to the American College of Medical Genetics and Genomics (ACMG) guidelines [23] as pathogenic, likely pathogenic, variant of unknown significance (VUS), likely benign, or benign [24, 25]. Only pathogenic, likely pathogenic, and VUS variants were reported for further analysis. All variants are reported using RefSeq transcript references (GRCh38/hg38).

### 2.4. Mutation Validation Methods

• Sanger sequencing. Candidate variants were confirmed by Sanger sequencing. Primers were designed using bioinformatics tools [26] and synthesized by Metabion (Germany). Sequencing was performed using the Big Dye Terminator v1.1 Kit (Thermo Fisher Scientific) on an ABI 3130/xl Genetic Analyzer. Data were analyzed with Gene Runner, Chromas, and NCBI‐BLAST.
• MLPA. Deletion/duplication variants detected by WES were confirmed using MLPA (MRC Holland) with SALSA MLPA Probemix P116‐B2 SGC for genes associated with LGMD (*SGCA*, *SGCB*, *SGCD*, *SGCG*, *FKRP*).
• STR haplotyping and segregation analysis. For selected families, variant segregation was assessed using STR haplotyping and linkage analysis, as previously described [14]. In this study, STR genotyping served two specific purposes: (i) to provide independent, locus‐specific validation of cosegregation by confirming that the disease phenotype tracks with the broader chromosomal region containing the candidate gene, thereby strengthening ACMG PP1 evidence, and (ii) to evaluate shared haplotypes among unrelated families from the same geographic regions, facilitating the assessment of potential founder effects in this highly consanguineous population. STR analysis was not used for pedigree verification or complex haplotype phasing.

**Figure 1:**
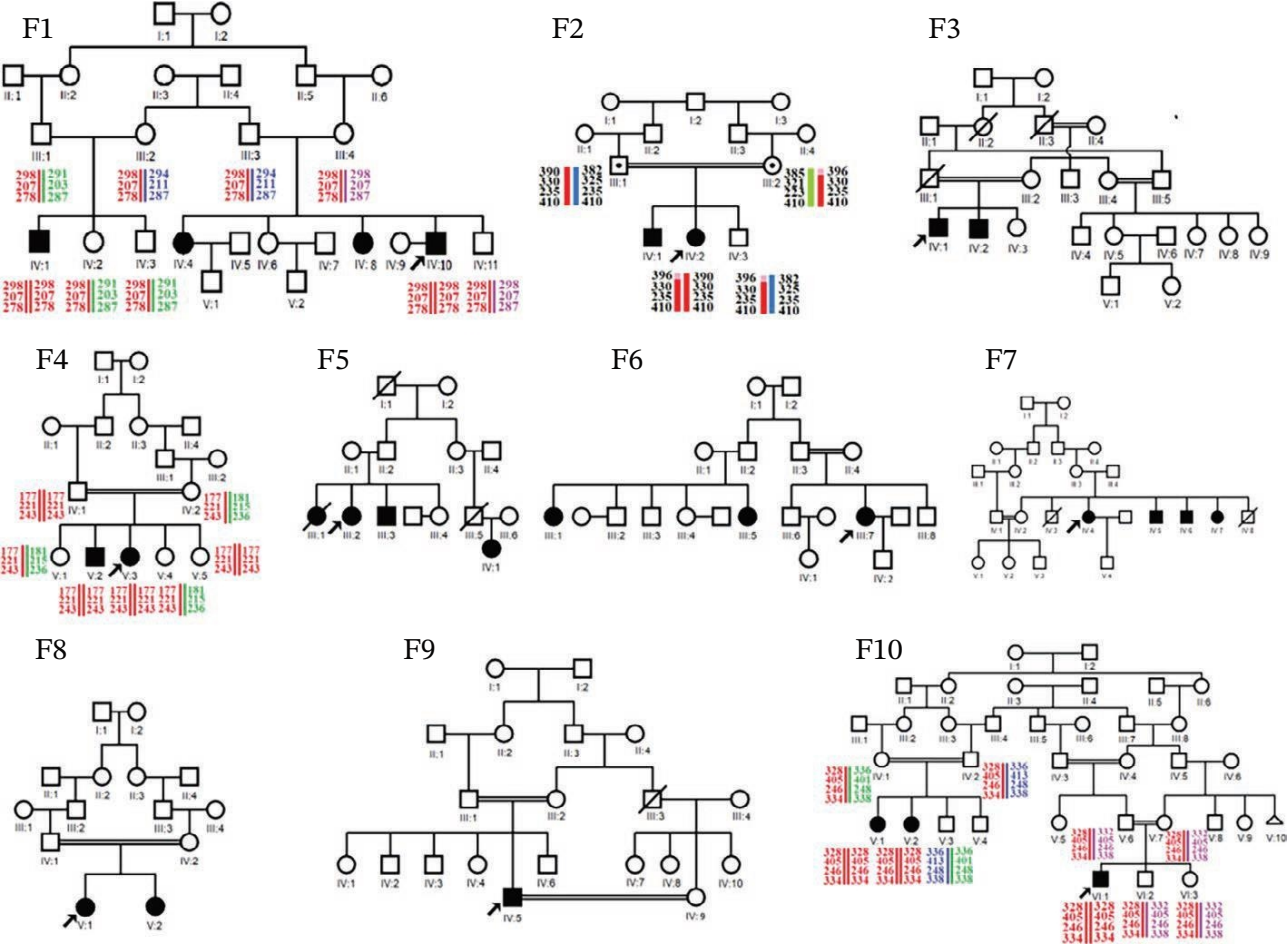
Pedigrees and STR haplotype analysis of the 10 studied families. Squares represent males, and circles represent females; filled symbols indicate affected individuals, open symbols indicate unaffected individuals, and symbols with a slash indicate deceased individuals. Double lines denote consanguineous marriages. Numerical annotations: Below each individual, the numbers represent the allele sizes (in base pairs) of the polymorphic STR markers analyzed. Each column of numbers corresponds to a specific STR marker flanking the candidate gene locus (ordered by genomic position). Although marker names are omitted for clarity, the columns represent the allelic configuration across the locus. Haplotype interpretation: Red numbers indicate the chromosomal segment (haplotype) that cosegregates with the disease phenotype. In autosomal recessive families (e.g., F1, F3, F4, and F10), affected individuals are homozygous for the red haplotype. The consistent red haplotype across affected family members confirms linkage between the marker locus and the disease gene.

Polymorphic STR markers flanking each candidate gene were selected based on their proximity to the gene (within a ±2‐Mb range) and a high heterozygosity index (> 0.7) in the local population. PCR amplification was performed using fluorescently labeled forward primers (6‐FAM, HEX, or NED) and unlabeled reverse primers. PCR products were pooled and analyzed by capillary electrophoresis on an ABI 3130xl Genetic Analyzer (Thermo Fisher Scientific) using GeneScan 500 LIZ size standard. Fragment sizes (allele sizes in base pairs) were determined using GeneMapper Software V5.0 (Thermo Fisher Scientific). Haplotypes were reconstructed manually and verified using Progeny V9 software (Progeny Genetics, United States) to track the inheritance of the disease‐associated chromosomal segment (represented by the red haplotype in Figure 1) across generations.

### 2.5. Statistical Analysis

Statistical analysis was performed using SPSS Statistics 22.0.0 (SPSS, Chicago, Illinois, United States).

### 2.6. AI‐Assisted Language Editing

During the preparation of this manuscript, our research team used AI‐assisted tools (e.g., ChatGPT and OpenAI) solely for language refinement and readability enhancement. We explicitly confirm that all scientific content, including the research design, methodology, data collection, analyses, interpretations, and conclusions, was independently developed by our team. The AI tools were not involved in generating scientific ideas, data, or interpretations, and their use was limited strictly to improving clarity, grammar, and presentation of the text.

## 3. Results

### 3.1. Overview of the Study

WES was performed on one affected individual from each of the 10 unrelated Iranian families, leading to the identification of 13 candidate disease‐causing variants. These included two pathogenic, four likely pathogenic, and six VUS, along with one large deletion. Among these, seven variants (54%) were novel and not previously reported in public databases. The main demographic and clinical characteristics of the 27 affected individuals are summarized in Table 1.

**Table 1:** Demographic and clinical features of studied patients.

Characteristics	Value
Patients	
Total	27
Gender	
Male *n* (%)	11 (40.7)
Female *n* (%)	16 (59.3)
Age	6–40 years
Age of onset	
Range	2–28 years
Mean	11.31 ± 7 years
Age of diagnosis	
Range	14–40 years
Mean	31 ± 9 years
Diagnosis delay	
Range	2–33 years
Mean	16.8 years
EMG with myopathic features	
Tested patients *n* (%)	13/27 (48)
Myopathic patients *n* (%)	13/13 (100)
Missing data *n* (%)	1/27 (3.7)
Muscle biopsy	
Tested patients *n* (%)	7/27 (26)
MD patients *n* (%)	6/7 (85.7)
Myopathic patients *n* (%)	1/7 (14.3)
Missing data *n* (%)	1/27 (3.7)
Ambulation status	
Wheelchair bound *n* (%)	13/27 (48)
With difficulty *n* (%)	4/27(14.8)
Normal *n* (%)	7/27 (25.9)
Missing data *n* (%)	3/27 (11)
Consanguinity *n* (%)	7/10 (70)

Consanguinity was reported in 70% (7/10) of families and 48% (13/27) of patients. For the remaining families, although the parents were not consanguineous, they were from the same region, suggesting possible distant relatedness or a shared founder effect. A positive family history consistent with autosomal recessive inheritance was present in 26 patients (96.3%), with only one sporadic case (F9). The mean age of onset was 11.31 ± 7 years, and the mean age at diagnosis was 31 ± 9 years, indicating a significant diagnostic delay of approximately 16.8 years.

All patients exhibited elevated serum CK levels. Detailed clinical and laboratory findings for each patient are presented in Table 2.

**Table 2:** Laboratory and clinical data of genetically diagnosed muscular dystrophy patients.

Family ID	Parents relativity^a^	PID	Sex	Age at onset/exam	CK (IU/L)	EMG with myopathic features/muscle biopsy	Signs and symptoms	Ambulation status^b^	Dystrophinopathies rolled out with^c^	Common LGMD testing results^d^
F1	A	IV‐1	M	28/33	NT	NT/NT	Muscle weakness, toe walking	A	A	No linkage
		IV‐4	F	17/40	↑	NT/NT	Muscle weakness, positive Gower sign, toe walking, scoliosis, lordosis	B		
		IV‐8	F	17/37	↑	NT/muscular dystrophy	Muscle weakness, positive Gower sign, toe walking, ankle contracture	B		
		IV‐10	M	18/36	↑	Myopathic/NT	Muscle weakness, positive Gower sign, toe walking, waddling gait, ankle contracture	B		

F2	B	IV‐1	M	8/26	661	Myopathic/NT	Muscle weakness, pseudohypertrophic muscles	B	B	No linkage
		IV‐2	F	8/24	1200	Myopathic/NT	Muscle weakness, scoliosis	B	B	

F3	B	IV‐1	M	—/35	3126	NT/NT	Muscle weakness	C	—	—
		IV‐2	M	10/33	2810	Myopathic/muscular dystrophy	Muscle weakness, dysphagia	C	B	NT

F4	B	V‐2	M	20/33	NT	NT/NT	Muscle weakness	A	A	No linkage
		V‐3	F	17/31	NT	Myopathic/muscular dystrophy	Muscle weakness, difficulty in running and climbing the stairs	A		

F5	A	III‐1	F	5/passed away	243	Myopathic/NT	Muscle weakness, movement limitation in shoulder girdle, positive Gower sign, scoliosis, tachycardia	B	A	No linkage
		III‐2	F	4/37	182	Myopathic/NT	Muscle weakness, positive Gower sign, ankle contracture, movement limitation in shoulder girdle, winging scapula	B		
		III‐3	M	3/19	603	N at 3 y/o/NT	Muscle weakness, positive Gower sign, ankle contracture, movement limitation in shoulder girdle, winging scapula, scoliosis	B		
		IV‐1	F	2/14	NT	Myopathic/muscular dystrophy	Muscle weakness, positive Gower sign, scoliosis	B		

F6	B	II‐1	F	10/39	NT	NT/muscular dystrophy	Muscle weakness, positive Gower sign, toe walking, scoliosis, lordosis, movement limitation in shoulder girdle, tachycardia	A	A	No linkage
		II‐5	F	10/28	712	Myopathic	Muscle weakness, positive Gower sign, winging scapula	C		
		II‐7	F	12/29	NT	NT/muscular dystrophy	Muscle weakness, positive Gower sign, winging scapula, tachycardia	C		

F7	A	IV‐4	F	19/33	NT	NT/NT	Muscle weakness	Dead	A	No linkage
		IV‐5	M	21/31	NT	NT/NT	Muscle weakness	A		
		IV‐6	M	—/29	↑	NT/NT	—	C		
		IV‐7	F	—/27	↑	NT/NT	Muscle weakness	A		

F8	B	V‐1	F	4/9	3490	Myopathic/myopathic	Muscle weakness	C	A	No linkage
		V‐2	F	4/6	3442	N at 2 y/o/NT	Muscle weakness	C		

F9	B	IV‐5	M	4/32	450	Myopathic/muscular dystrophy	Muscle weakness, repeated falling, toe walking	B	B	—

F10	B	V‐1	F	—/—	2000	Myopathic/NT	Muscle weakness, positive Gower sign	B	A	—
		V‐2	F	—/—	11,140	Myopathic/NT	Muscle weakness, positive Gower sign	B		
		VI‐1	M	8/24	2000	Myopathic/muscular dystrophy	Muscle weakness, positive Gower sign	B		

*Note:* — represents missing data.

Abbreviation: NT, not tested.

^a^A, from the same region; B, consanguine.

^b^A, with difficulty; B, wheelchair bound; C, normal.

^c^A, affected female; B, negative DDx gene deletion in molecular methods.

^d^Common LGMD testing results: Refers to pre‐WES targeted molecular investigations, including linkage analysis and Sanger sequencing of common LGMD‐associated genes. “No linkage” indicates that linkage analysis did not identify a shared homozygous region consistent with the disease phenotype.

EMG revealed myopathic features in all 13 tested patients. Muscle biopsy showed dystrophic changes in six of seven examined cases, with one showing myopathic but not dystrophic features. Ambulation status varied, with 48.1% wheelchair bound, 14.8% experiencing difficulty walking, and 25.9% having normal ambulation.

Prior to WES, all families had undergone conventional genetic testing (e.g., linkage analysis, MLPA, and Sanger sequencing), which ruled out dystrophinopathies (DMD/BMD) and common LGMD gene mutations. Despite these efforts, the molecular cause remained unknown.

### 3.2. WES Findings and Mutation Confirmation

WES led to the identification of 13 candidate variants across 10 families. The detailed characteristics of these variants are presented in Table 3. Among the 13 variants, seven (54%) were novel; six were missense, two frameshift, two splice‐site, one stop‐gained, one synonymous, and one large deletion; six were classified as pathogenic or likely pathogenic (46.2%), and seven were VUS (53.8%).

**Table 3:** Characteristics of the genetic mutations found in the genetically diagnosed muscular dystrophy patients.

Family number	Gene	Chromosomal position (hg38)	Mutation^a^	OMIM	ACMG classification^b^	In silico prediction	ClinVar reports	Complementary tests^c^
F1	DYSF	Chr 2: 71503308	NM_001130979.1:c.331C>T, p.Gln111Ter^(A1)^	AR,#253601	P	CADD phred (39), GERP (4.96)	Pathogenic, criteria provided, multiple submitters, no conflicts	A and B
F2	SGCA	Chr 17: 50168466	NM_000023.4:c.477‐478insC, p.Ala160ArgfsTer31^(A2)^	AR,#608099	LP	—	Not reported	A and B
F3	TK2	Chr 16: 66531424	NM_004614.5:c.331A>G, p.Thr111Ala^(A3)^	AR,#609560	VUS	CADD phred (25.2), GERP (5.95), SIFT (0.01, damaging), Revel (0.65, deleterious)	Not reported	A
				AR,#617069				
	LAMA2	Chr 6: 129353336	NM_001079823.1:c.4696C>T, p.Arg1566Cys^(A3)^	AR,#607855	VUS	CADD phred (23.3), GERP (4.88), SIFT (0.03, damaging), Revel (0.2, benign)	Uncertain significance, criteria provided, single submitter	A
				AR,#6181138				
F4	MAP3K20	Chr 2: 173203795	NM_016653.3:c.670‐1G>T^(A4)^	AR,#617760	LP	CADD phred (32), GERP (5.82), SpliceAI (0.97, splice‐altering), dbscSNV Ada (1, deleterious), RF (0.93, deleterious)	Not reported	A and B
F5	LMNA	Chr 1: 156135197	NM_170707.4:c.821C>T, p.Ala274Val^(A3)^	AR,#616516	VUS	CADD phred (28.8), GERP (4.38), SIFT (0.03, damaging), Revel (0.72, deleterious)	Not reported	A
				AR,#605588				
F6	COL6A1	Chr 21: 45987653	NM_001848.2:c.803A>G, p.Glu286GLY^(A3)^	AD/AR,#158810	VUS	CADD phred (29.3), GERP (3.92), SpliceAI (0, benign), dbscSNV Ada (0.99, deleterious), RF (0.91, deleterious), SIFT (0.01, damaging), Revel (0.52, deleterious)	Not reported	A
				AD/AR,#254090				
	COL6A2	Chr 21: 46131981	NM_001849.3:c.2489G>A, p.Arg830Gln^(A3)^		VUS	CADD phred (29.3), GERP (4.18), SIFT (0.14, tolerated), Revel (0.62, deleterious)	Conflicting interpretations of pathogenicity, criteria provided	A
F7	DYSF	Chr 2: 71569898	NM_001130979.2:c.2982C>T, p.Gly994=^(A5)^	AR,#253601	VUS	CADD phred (16.96), SpliceAI (0.92, splice‐altering)	Uncertain significance, criteria provided, single submitter	A
	ITGA7	Chr 12: 55685189	NM_001144996:c.3294_3295insGT, p.Lys1099SerfsTer7^(B2)^	AR,#613204	LP	—	Not reported	
F8	MICU1	Chr 10: 72368337	NM_006077.3:c.1294‐1295delT, p.Asn432Ilefs ^∗^8^(A2)^	AR,#615673	LP	—	Not reported	A
F9	COL6A2	Chr 21: 46117454	NM_001849.3:c.1053+1G>T^(B6)^	AD/AR,#158810	P	CADD phred (34), GERP (5.03), SpliceAI (0.99, splice‐altering), dbscSNV Ada (1, deleterious), RF (0.93, deleterious)	[27, 28]	A
F10	SGCB	Chr 13	—^(A7)^	AR,**#**604286	LP		[29, 30]	B and C

^a^A, homozygote state; B, heterozygote state; 1, stop‐gained; 2, frameshift; 3, missense; 4, splice_acceptor; 5, synonymous; 6, substitution; 7, large deletion.

^b^P, pathogenic; LP, likely pathogenic; VUS, variant of uncertain significance.

^c^Complementary tests: Methods used for validation and segregation analysis of WES‐identified variants. (A) Sanger sequencing: Confirmation of point mutations/small indels; (B) STR haplotyping/linkage analysis: Assessment of variant segregation within families; (C) MLPA: Confirmation of copy number variants (deletions/duplications).

**Figure 2:**
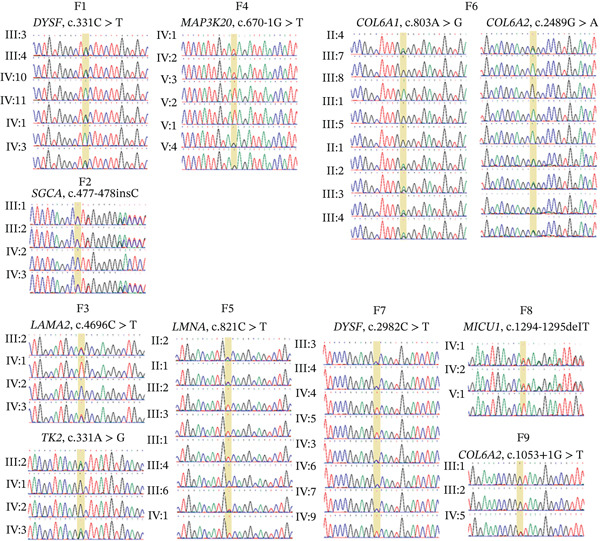
Sanger sequencing chromatograms confirming disease‐causing variants in selected family members.

**Figure 3:**
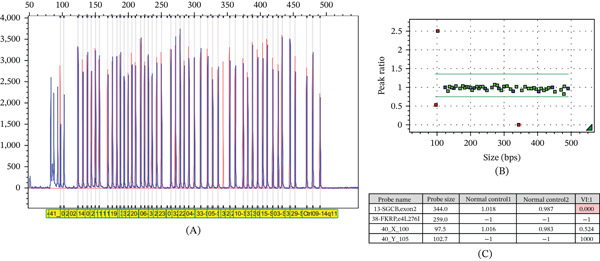
MLPA analysis of Family 10 proband: (A) panel marker calling and alignment, (B) peak ratio graph, and (C) MLPA probes characteristics. The results of this test show a deletion in exon 2 of the *SGCB* gene.

**Figure 4:**
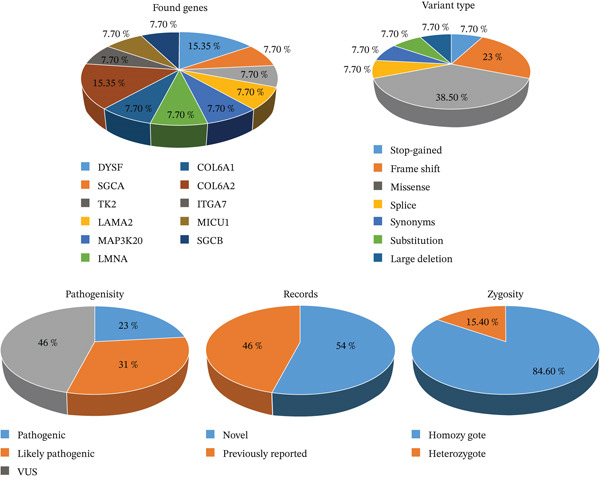
Pie charts of identified genes and variations. The charts show the distribution of variants based on pathogenicity (pathogenic, likely pathogenic, VUS), novelty (novel vs. known), and zygosity (homozygous vs. heterozygous).

The hereditary pattern was autosomal recessive in 84.6% of cases, with patients being homozygous for the identified variants. One case (F9) showed an autosomal dominant pattern due to a de novo mutation. The family pedigrees and STR haplotyping results are shown in Figure 1. Sanger sequencing confirmed the presence of candidate variants in affected individuals and their segregation within families (Figure 2). For Family 10, MLPA analysis confirmed a homozygous deletion in exon 2 of the *SGCB* gene (Figure 3). The distribution of variants based on pathogenicity, novelty, and zygosity is summarized in Figure 4.

### 3.3. Challenging Cases and Key Findings

In Family 3, two homozygous missense variants were identified in *TK2* (c.331A>G, p.Thr111Ala) and *LAMA2* (c.4696C>T, p.Arg1566Cys). Sanger sequencing revealed that the affected brother did not carry the *LAMA2* variant, excluding it as causative. The *TK2* variant showed cosegregation with the disease and was reclassified as likely pathogenic based on ACMG criteria (PP1 added). This finding supports a diagnosis of mitochondrial DNA depletion syndrome (MDDS).

In Family 4, a novel homozygous splice‐site variant in *MAP3K20* (c.670‐1G>T) was identified. Initially, Individual IV‐5 was considered unaffected, but Sanger sequencing revealed she was homozygous for the variant. Follow‐up confirmed she had developed muscle weakness, confirming her as an affected individual. This highlights the importance of long‐term follow‐up in presymptomatic carriers.

In Family 6, no definitive pathogenic variant was found after initial filtering. However, two homozygous missense variants were identified in *COL6A1* (c.803A>G, p.Glu286Gly) and *COL6A2* (c.2489G>A, p.Arg830Gln). Given the close proximity of these genes on chromosome 21 and strong linkage disequilibrium, it is likely they are coinherited. Functional studies are needed to determine which variant, if any, is primarily responsible for the phenotype.

In Family 7, a homozygous synonymous variant in *DYSF* (c.2982C>T, p.Gly994=) was identified. Despite being synonymous, it is predicted to alter splicing (SpliceAI = 0.92) and is absent from population databases. This case underscores that synonymous variants should not be automatically dismissed as benign. Additionally, a heterozygous frameshift variant in *ITGA7* was found, classifying the proband as a carrier. Genetic counseling was recommended due to the risk of future affected offspring in consanguineous unions.

In Family 9, a heterozygous splice‐site variant in *COL6A2* (c.1053+1G>T) was identified. The variant was absent in both parents, indicating a de novo mutation. This confirms an autosomal dominant inheritance pattern, consistent with Bethlem myopathy.

## 4. Discussion

In this study, we applied WES to identify the genetic basis of nondystrophinopathic MDs in 10 Iranian families with unresolved diagnoses. Our approach led to the identification of 13 candidate variants in 10 known MD‐associated genes, including seven novel mutations. The diagnostic yield of WES was 69.2% after segregation analysis and ACMG‐based reclassification, highlighting the power of this method in resolving genetically heterogeneous and diagnostically challenging cases.

Our findings underscore the genetic diversity of MDs in Iran, particularly in the context of high consanguinity (70% of families). The predominance of autosomal recessive inheritance (84.6%) aligns with previous reports from consanguineous populations, where homozygous variants are more common due to shared ancestral haplotypes. The identification of a de novo variant in *COL6A2* in a sporadic case (F9) also emphasizes the importance of considering autosomal dominant forms, even in the absence of family history.

Among the most notable findings was the identification of two homozygous missense variants in *COL6A1* (c.803A>G) and *COL6A2* (c.2489G>A) in Family 6. These genes are located in close proximity on chromosome 21 and are known to be in strong linkage disequilibrium, particularly in populations with founder effects. This genetic architecture makes it difficult to determine which variant, if any, is primarily responsible for the phenotype. Both genes encode components of collagen VI, and pathogenic variants in either can lead to overlapping phenotypes, including Bethlem myopathy and Ullrich congenital MD. In silico predictions suggest both variants are deleterious; however, functional studies are required to elucidate their individual and combined effects on collagen VI assembly and extracellular matrix integrity.

The discovery of a novel splice‐site variant in *MAP3K20* (c.670‐1G>T) in Family 4 is particularly significant. This gene has recently been associated with autosomal recessive limb‐girdle muscular dystrophy type 29 (LGMD R29). The reclassification of an initially “unaffected” family member (IV‐5) as presymptomatic after genetic testing highlights the variable expressivity and age‐dependent penetrance of some MDs. This finding has critical implications for genetic counseling and underscores the necessity of long‐term clinical follow‐up for at‐risk individuals, even in the absence of current symptoms.

We also identified a synonymous variant in *DYSF* (c.2982C>T) classified as VUS, which is predicted to alter splicing (SpliceAI = 0.92). This case reinforces the growing evidence that synonymous variants should not be automatically dismissed as benign, as they can disrupt splicing regulatory elements, mRNA stability, or translational kinetics.

The high proportion of novel variants (54%) in our cohort reflects the underrepresentation of Middle Eastern populations in global genomic databases. This highlights the importance of population‐specific genetic studies to enrich reference databases and improve variant interpretation accuracy for non‐European populations.

A striking finding in our study was the significant intrafamilial phenotypic variability observed in Family 1, despite all affected members carrying the same homozygous nonsense variant in *DYSF* (c.331C>T, p.Gln111Ter). Although most affected individuals (e.g., IV‐10, IV‐4, and IV‐8) presented with classic early‐onset symptoms (ages 12–17 years) and rapid progression to wheelchair dependence, Individual IV‐1 exhibited a markedly delayed onset at 28 years and retained ambulation with assistance at age 31. This discrepancy highlights that identical genotypes do not always yield uniform phenotypes in dysferlinopathies.

The p.Gln111Ter variant truncates the protein in the N‐terminal FerA domain, essential for membrane binding. Although truncating variants in this domain are generally associated with severe LGMD2B, the late onset in IV‐1 suggests the influence of modifying factors. Potential contributors include (1) genetic modifiers, such as polymorphisms in genes involved in alternative membrane repair pathways (e.g., *ANO5* and *MG53*) or inflammatory regulation, which may mitigate muscle damage; (2) environmental factors, including differences in physical activity levels and occupational demands during critical developmental periods; and (3) epigenetic modifications, such as differential DNA methylation or miRNA expression, which may alter residual gene expression or compensatory mechanisms. Additionally, stochastic variations in muscle regeneration capacity may play a role. This case underscores the importance of considering nongenetic factors in prognosis and highlights the limitations of predicting disease trajectory based solely on genotype. It also emphasizes the need for long‐term monitoring of at‐risk family members, as late‐onset presentations can occur even in families with historically early‐onset case [20].

Detailed clinical phenotypes corresponding to the novel variants identified in this study are summarized in Table 2. Specifically, the novel *DYSF* nonsense variant (p.Gln111Ter) in Family 1 was associated with a remarkably variable age of onset among affected siblings (ranging from 17 to 28 years), with Individual IV‐1 presenting late‐onset weakness at age 28. The novel frameshift variant in *SGCA* (p.Ala160ArgfsTer31) in Family 2 correlated with a severe, early‐onset phenotype, with symptoms appearing at age 8 and rapid progression to wheelchair dependence. The novel *TK2* missense variant (p.Thr111Ala) in Family 3 was linked to proximal muscle weakness and dysphagia starting at age 10, consistent with the myopathic form of mitochondrial depletion syndrome. Similarly, the novel *MAP3K20* splice‐site variant in Family 4 was associated with onset in late adolescence (age 17). These correlations reinforce the genotype–phenotype landscape of these genes and highlight the utility of WES in capturing the full clinical spectrum, including variable expressivity and atypical onsets.

To elucidate the molecular consequences of the variants identified in this cohort, we mapped each variant to its corresponding protein domain using UniProt and Pfam databases. Loss‐of‐function variants in *DYSF* (p.Gln111Ter) and *SGCA* (p.Ala160ArgfsTer31) are predicted to trigger nonsense‐mediated decay, resulting in complete absence of dysferlin and *α*‐sarcoglycan—proteins essential for sarcolemmal repair and dystrophin–glycoprotein complex stability, respectively. The *TK2* p.Thr111Ala missense variant localizes to the enzyme′s catalytic domain, likely impairing mitochondrial dNTP salvage and explaining the MDDS phenotype. The *MAP3K20* c.670‐1G>T splice‐site variant is predicted to induce exon skipping and NMD, expanding the mutational spectrum of the recently described LGMD R29. The *LMNA* p.Ala274Val variant resides in the critical *α*‐helical rod domain, disrupting nuclear lamina integrity consistent with laminopathy phenotypes. Both *COL6A1* p.Glu286Gly and *COL6A2* p.Arg830Gln map to the triple‐helical collagenous domain; substitution of charged residues in this structurally constrained region likely disrupts microfibril assembly, though strong linkage disequilibrium between these tightly linked genes complicates attribution of pathogenicity. The synonymous *DYSF* p.Gly994= variant, predicted to alter splicing (SpliceAI = 0.92), underscores that synonymous changes should not be dismissed without functional assessment. The *MICU1* frameshift variant likely causes NMD and mitochondrial calcium dysregulation, adding to the emerging MICU1‐related myopathy phenotype. Finally, the de novo *COL6A2* splice‐site variant confirms autosomal dominant Bethlem myopathy in a sporadic case, whereas the *SGCB* exon 2 deletion replicates an Iranian founder mutation. Collectively, these findings expand our understanding of MD pathogenesis by confirming domain‐specific vulnerability, demonstrating the pathogenic potential of splice‐altering variants, and providing population‐specific genotype–phenotype correlations.

To contextualize our findings within the existing literature, we compared the genomic position and clinical severity of our novel variants with previously reported mutations in the same genes. This comparative analysis is summarized in Table S1. As shown, truncating variants in critical domains such as the FerA domain of *DYSF* and the rod domain of *LMNA* consistently correlated with severe, early‐onset phenotypes in our cohort, aligning with global reports [31–37]. However, notable intrafamilial variability was observed, particularly in Family 1, suggesting the influence of modifier factors.

Although Sanger sequencing remains the gold standard for confirming specific nucleotide variants, we incorporated STR haplotyping to provide orthogonal validation of locus linkage and to assess shared ancestral haplotypes in our consanguineous cohort. STR analysis independently confirmed that the disease phenotype cosegregated with the chromosomal region harboring the candidate variant, thereby strengthening segregation evidence (ACMG PP1) without relying solely on single‐nucleotide tracking. Furthermore, given the geographic clustering and high consanguinity rate (70%) in our population, STR haplotyping enabled the identification of shared disease‐associated haplotypes among unrelated families from similar regions. This approach supports the identification of founder mutations, which is particularly relevant in Iranian populations where historical isolation and endogamy have preserved specific pathogenic alleles (e.g., the *SGCB* exon 2 deletion in Family 10). Importantly, STR analysis in this study was not employed for pedigree verification or complex phasing; its application was strictly confined to linkage validation and founder‐effect assessment, offering a targeted, cost‐effective complement to WES and Sanger sequencing.

Despite its strengths, this study has limitations. First, WES was performed on a single affected individual per family, which may have missed complex structural variants or deep intronic mutations. Second, functional validation (e.g., RNA sequencing and protein expression) was not performed for most variants due to sample unavailability. Third, the small sample size limits the generalizability of our findings, though it is comparable to similar WES studies in rare diseases.

## 5. Conclusion

In conclusion, our study demonstrates that WES is a highly effective tool for diagnosing undiagnosed MDs, particularly in consanguineous populations. The identification of novel and rare variants expands the mutational spectrum of MDs in Iran and provides valuable data for genetic counseling, prenatal diagnosis, and future therapeutic strategies. Future studies should incorporate RNA‐seq and functional assays to confirm pathogenicity and explore genotype–phenotype correlations in larger cohorts.

## Author Contributions

N.S. and S.Z. made substantial contributions to the conception of the work; N.S., S.Z., S.D.B., and M.K. contributed to the design of the work; F.Z.M., S.A., S.Y., and H.B. were involved in the acquisition of data; N.S., M.S.F., T.S., and G.R.S. participated in the analysis and interpretation of data; Z.M., N.S., S.Z., Z.S., and R.Z. drafted the work and/or substantively revised it.

## Funding

No funding was received for this manuscript.

## Disclosure

All authors commented on the manuscript, read, and approved the final manuscript.

## Ethics Statement

Prior to sampling, genetic counseling was performed, and informed consent from the family was obtained. The project was approved by the Ethical Committee of the Pasteur Institute of Iran (No: 91/0201/10425).

## Consent

The authors have nothing to report.

## Conflicts of Interest

The authors declare no conflicts of interest.

## Supporting information


**Supporting Information** Additional supporting information can be found online in the Supporting Information section. Table S1: Detailed comparison of novel variants with previously reported mutations by position and clinical severity.

## Data Availability

The data that support the findings of this study are available from the corresponding author upon reasonable request.
