# Supplementary material for: Whole‐Exome Sequencing in Undiagnosed Muscular Dystrophies: A High Diagnostic Yield and Novel Insights From Iranian Families
Source: Hum Mutat. 2026 Aug 3;2026:6592066. doi: 10.1155/humu/6592066 (PMC13434219; doi:10.1155/humu/6592066)
Supplement: Supplementary file 1 [file HUMU-2026-6592066-s001.docx]

**Supplementary Table S1:** Detailed Comparison of Novel Variants with Previously Reported Mutations by Position and Clinical Severity

| **Gene** | **Variant (This Study)** | **Protein Domain/Position** | **Previously Reported Variants in Similar Regions** | **Typical Clinical Severity (Literature)** | **Clinical Severity (Our Cohort)** | **Interpretation/Notes** |
| --- | --- | --- | --- | --- | --- | --- |
| DYSF | c.331C>T (p.Gln111Ter) | N-terminal C2A/FerA domain | Truncating variants in exons 4–10 | Early onset (<15 yrs), rapid progression to wheelchair by late teens(31) | Onset 17–28 yrs; wheelchair by 18–28 yrs | Aligns with severe LOF phenotype; intrafamilial variability suggests modifier effects |
| SGCA | c.477_478insC (p.Ala160ArgfsTer31) | Transmembrane/extracellular domain (Exon 5) | Frameshifts in exons 4–6 | Severe early-onset sarcoglycanopathy; loss of ambulation by adolescence(30) | Onset 8 yrs; wheelchair-dependent by age 8 | Matches classic severe early-onset phenotype |
| MAP3K20 | c.670-1G>T | Splice acceptor (Intron 8) | Few reported; mostly missense/LOF in LGMD R29 | Variable; some LOF variants show childhood onset(32, 33) | Onset 17–20 yrs; ambulatory with difficulty at 30s | Expands mutational spectrum; later onset suggests possible residual function or modifiers |
| LMNA | c.821C>T (p.Ala274Val) | Coil 2B/α-helical rod domain | Missense variants in rod domain (aa 250–300) linked to LGMD1B/EDMD | Childhood/adolescent onset, progressive weakness, frequent cardiac involvement(34) | Onset 3–5 yrs; wheelchair by 30s; tachycardia documented | Consistent with severe LMNA-related myopathy spectrum |
| COL6A1/2 | p.Glu286Gly / p.Arg830Gln | Triple-helical collagenous domain | Missense variants here cause Bethlem (milder) or Ullrich CMD (severe) | Ullrich: onset <2 yrs, early contractures. Bethlem: later onset, preserved ambulation(35) | Onset 10–12 yrs; ambulatory with difficulty/normal; no severe contractures | Phenotype aligns closer to Bethlem/milder spectrum; highlights position-dependent severity modulation |
| DYSF | c.2982C>T (p.Gly994=) | C2D domain (synonymous) | Synonymous variants rarely reported as pathogenic | N/A | Onset ~19–21 yrs; progressive proximal weakness | First report of a splice-altering synonymous variant in DYSF; expands variant interpretation guidelines |
| MICU1 | p.Asn432Ilefs*8 | Mitochondrial calcium uptake regulator | Frameshifts leading to NMD | Myopathy with mitochondrial dysfunction features(36) | Mild weakness, elevated CK, normal ambulation at age 9 | Adds to emerging MICU1-related myopathy phenotype |
| COL6A2 | c.1053+1G>T | Splice donor (Intron 11) | Canonical splice-site variants in COL6A2 | Autosomal dominant Bethlem myopathy(37) | Onset 4 yrs; toe walking, proximal weakness | Confirms de novo AD mechanism in sporadic case |
| SGCB | Exon 2 deletion | Structural exon of β-sarcoglycan | Large deletions in SGCB | Severe LGMD2E phenotype (29) | Onset 8 yrs; wheelchair-bound by 15 yrs | Replicates Iranian founder mutation; reinforces LOF paradigm |
